# Age Moderates the Effect of Awe on Cognitive but not Emotional Well-Being

**DOI:** 10.1093/geroni/igaa057.1473

**Published:** 2020-12-16

**Authors:** Laura Bernstein, Julie Patrick

**Affiliations:** West Virginia University, Morgantown, West Virginia, United States

## Abstract

Evidence suggests that positive emotions may broaden and build our emotional and physical health, and cognitive resources (Fredrickson, 2001). A growing literature shows that happiness and joy can be powerful means for growth. In contrast to happiness, which pushes one to expand and accommodate, research suggests that awe, that feeling of being in the presence of something immense or transcendent, prompts the urge to assimilate. Although promising, few examinations have included older adults and a limited range of positive emotions have been examined. Thus, we sought to address this gap in the literature by assessing the influence of age and awe on emotional well-being. Data from 180 adults (M age ~ 38; range 18 – 89) were used to examine the main effects of age and dispositional awe (Shiota et al., 2006), and their interaction, on emotional well-being. Only 12% of the variance was explained [X2 (DF = 9) = 344.27, p < .001]. Awe was positively associated with emotional well-being (Beta = .280*), but neither age nor the interaction between age and awe contributed to the variance explained. We conducted a similar examination with perceived cognitive health [X2 (DF=9) = 337.09, p < .001; R^2 = .235]. A main effect for age and a significant age by awe interaction uniquely contributed to the variance explained in cognitive well-being. A similar model was tested with self-assessed health as the outcome. Neither main effects nor the interaction emerged as significant. Results are discussed within the context of age-invariant contributors to well-being.

